# A service evaluation of virtual wards in Cornwall, UK

**DOI:** 10.1093/oodh/oqaf008

**Published:** 2025-04-14

**Authors:** Helen Lyndon, Tracey Viney, Vicki Slade

**Affiliations:** University of Plymouth, South West Clinical School, Drake Circus, Plymouth, Devon, PL4 8AA, UK; Cornwall Partnership NHS Foundation Trust, Digital Health Service, Bodmin, Carew House, Beacon Technology Park, Dunmere Road, Bodmin, PL31 2QN, Cornwall, UK; Marjon University, School of Health and Wellbeing, Derriford Road, Plymouth, PL6 8BH, Devon, UK; Cornwall Partnership NHS Foundation Trust, Digital Health Service, Bodmin, Carew House, Beacon Technology Park, Dunmere Road, Bodmin, PL31 2QN, Cornwall, UK

**Keywords:** digital health, virtual ward, service evaluation, community healthcare

## Abstract

This service evaluation provides an overview of the virtual ward model in Cornwall, UK, and was implemented using the Consolidated Framework for Implementation Research (CFIR). Interviews were conducted with virtual ward patients and clinicians and analysed using thematic analysis. A virtual ward is a digitally enabled service enabling people requiring hospital-level care to receive that care at home, either as an alternative to hospital admission or by facilitating an earlier discharge. Four themes emerged from the data: (i) Readiness for change: the virtual ward service was not embedded in existing health provision with scepticism and reluctance to refer to the virtual ward. (ii) Confidence and trust: due to system incompatibility issues, clinicians lost confidence and trust in the remote monitoring system; however, patients had high levels of trust in the virtual ward staff, increasing their confidence to remain at home. (iii) Digital challenges: using the monitoring equipment was challenging for some patients with issues of digital exclusion including understanding the technology and connection difficulties. (iv) Impact: despite the challenges, the virtual ward was highly valued by patients and supports person-centred care, offering a safe alternative to hospital admission. Virtual wards in Cornwall were rapidly implemented leading to some implementation barriers; nonetheless, the overwhelming response from patients demonstrated how they valued the virtual ward as a viable alternative to hospital admission and how the compassion and professionalism shown by the virtual ward clinicians made them feel safe and supported in their own homes.

## INTRODUCTION

Virtual wards have been part of healthcare provision for several decades; first introduced in Croydon, UK, in 2006, they provided intensive, multi-professional care using the methods, staffing and regular practices of a hospital ward [1]. In the two decades since their introduction, virtual wards have evolved to include some form of remote digital health interventions such as biometrics monitoring and virtual consultations. In 2023, the World Health Organization (WHO) produced a classification of digital health interventions, services and applications in health [2]. Within this classification, virtual wards are positioned within telemedicine, providing healthcare services at a distance. This includes the use of equipment providing remote consultations between people and healthcare providers and the remote monitoring of a person’s health and diagnostic data. The virtual ward model was adopted internationally in countries such as the USA, Australia and Canada and developed rapidly during the COVID-19 pandemic as an alternative to hospital admission. These virtual wards encompassed technology as part of the care offered, providing pulse oximetry and monitoring for patients with COVID-19, and now, most virtual wards offer a technology-enabled service with remote monitoring of patients supported by digital technology platforms [3]. A recent definition of a virtual ward is:

‘a time-limited service enabling people who have an acute condition or exacerbation of a chronic condition requiring hospital-level care to receive this care in the place they call home, either as an alternative to hospital admission or by facilitating an earlier discharge from hospital’ [4].

In recent years, particularly in the UK, virtual wards have multiplied in number and form within a context of existing longstanding telehealth or other digital health services in some health settings.

In Cornwall and the Isles of Scilly, UK, a digital health service has been in operation for over 15 years, managed by the community health services provider, Cornwall Foundation NHS Trust (CFT). Initially, it supported people with stable, long-term respiratory and cardiac conditions who were at risk of deterioration. The service proactively used remote monitoring to identify deterioration and to escalate to primary health care services for intervention. Since 2020, digital health services have transformed locally and nationally and are embedded in the operational post-pandemic recovery plans of health systems. Digital health in the area has developed in two directions; the first, virtual care for people with long term conditions who would benefit from remote monitoring but not as an alternative to bedded care, and the second, virtual wards for people who require enhanced health care at home as an alternative to a hospital bed utilizing, where appropriate, remote monitoring/hybrid models of face to face and remote care under the remit of NHS(National Health Service)@home. Two virtual wards have been established in the area: one for patients with respiratory illnesses and another for those with frailty. These wards aim to meet the NHS England requirement of providing a minimum of 40−50 beds per 100 000 population. Virtual wards are now viewed as a vital service within the overall healthcare provision in Cornwall to provide increased system capacity to care for patients at home as an alternative to inpatient admission. Currently, this extra capacity enables over 300 people a month to receive treatment at home, saving over 2700 inpatient bed days each month.

## EVALUATION PURPOSE

Prior to 2023, Cornwall digital health services had used an intermittent monitoring solution in both the virtual care and virtual ward services, primarily because there were no other available alternatives. In 2023, funding was provided to undertake a 6-month pilot of continuous monitoring with wearable devices to inform further service development and future procurement of remote monitoring systems. The difference between the two types of systems is that when using intermittent monitoring, the patient must put on and take off the monitoring devices at an agreed time for biometric readings to be taken and sent to the virtual ward team. With continuous monitoring systems, the patient has a wearable device, such as an arm cuff, that is worn continuously. The device records biometrics and transmits to the virtual ward team via a digital platform on a routine basis. This is a new development with limited evaluation of the types and benefits of technology used for remote patient monitoring. To ensure there was a robust academic evaluation of the continuous monitoring pilot project, NHS England and CFT commissioned this service evaluation which was conducted by the University of Plymouth South West Clinical School.

The procurement process for continuous monitoring equipment began in January 2023, and the pilot project was planned to go live on 1 May 2023; however, procurement was delayed, and the continuous monitoring equipment was not in use within the virtual ward until August 2023. Unfortunately, due to incompatibility with existing systems and associated clinical safety concerns, intermittent monitoring was discontinued after only a few weeks of use. This meant that the service evaluation could not fully fulfil its original aim of comparing the use of continuous and intermittent monitoring in a virtual ward setting. After discussion with the service evaluation steering group, it was agreed that the aim of the project would evolve to provide an overview of the virtual ward model in Cornwall including the lessons learned from the continuous monitoring pilot. This would provide intelligence to inform future procurement of a permanent digital health monitoring system.

## MATERIALS AND METHODS

### Evaluation design

**Figure 1:**
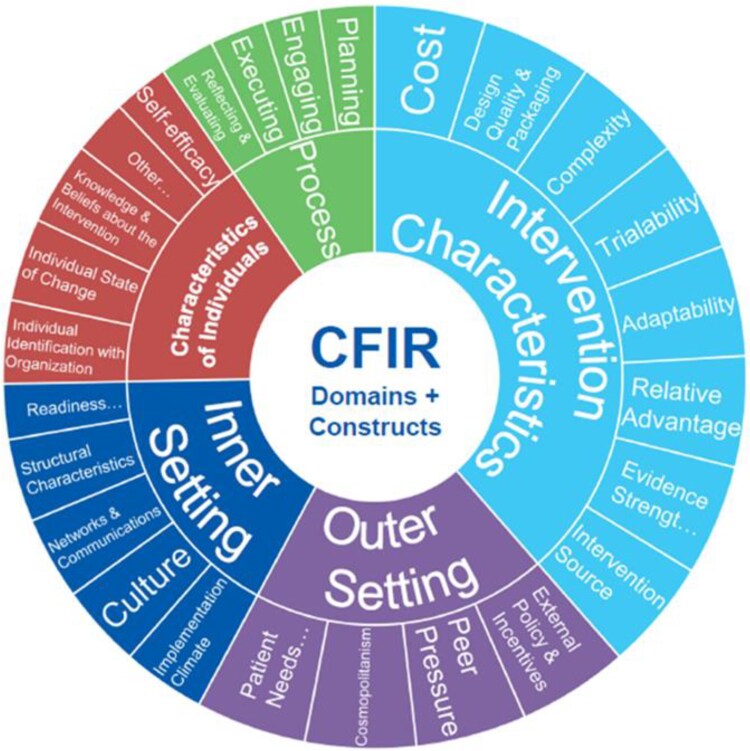
The consolidated framework for implementation research

The service evaluation was conducted using the Consolidated Framework for Implementation Research (CFIR) [5]. CFIR is a ‘meta-theoretical framework that provides a repository of standardized implementation-related constructs that can be applied across the spectrum of implementation research’ [6]. It comprises 26 constructs organized across five domains, all of which interact to influence the implementation of an intervention, implementation effectiveness and outcomes (Fig. 1). CFIR is used in implementation science research and evaluation to promote theory development and confirmation of what works, where and why across multiple circumstances in healthcare. Constructs can be selected that are most relevant to the project and its setting to guide evaluation of the implementation processes and implementation progress and to help explain findings [5]. A steering group was established comprising digital health clinicians and managers, academics and a person with lived experience, to guide the evaluation process.

The evaluation followed COREQ guidelines to strengthen credibility [17]. The researchers acknowledged that their backgrounds and values could influence the findings. Hence the steering group with varied expertise guided the process, ensuring balanced interpretations. Potential biases were addressed through triangulation discussions. Reflective logs were used to document decisions and biases to aid researchers with transparency and validity.

### Interview data collection

**Figure 2:**
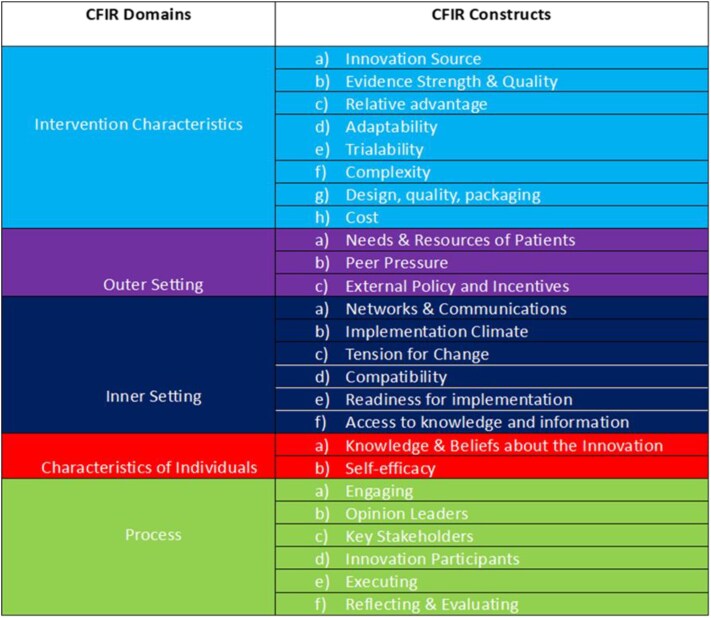
Coding framework

A deductive approach was taken to data collection with a coding framework created using the CFIR domains and constructs relevant to the evaluation (Fig. 2). The CFIR domains and constructs were shared with the service evaluation steering group, and relevant constructs were selected to meet the evaluation aims. Interview topic guides were created using these CFIR domains and constructs (Supplementary Files 1 and 2). Interviews were conducted with 14 patients and 16 clinicians to gain their opinions and feedback on being part of the virtual wards and their experiences with the different types of monitoring. Interviews were semi-structured, so the number of questions varied according to the flow of the interview, driven by the topic guide questions and prompts. Interviews lasted ~30 minutes (patient) and 40 minutes (clinician). Interview data were transcribed verbatim. Data were analysed using thematic analysis [7] by two team members, and their coding was verified by another team member who was familiar with the digital health service but was not involved in data capture, thereby reducing the risk of unconscious or conscious bias. Findings were presented within the CFIR constructs and shared with the steering group, enabling further verification and refinement.

### Ethical approval

Ethical approval was not required as this project was assessed as a service evaluation, not a research project using the UK National Health Service Health Research Authority decision tool.

## RESULTS

**Figure 3:**
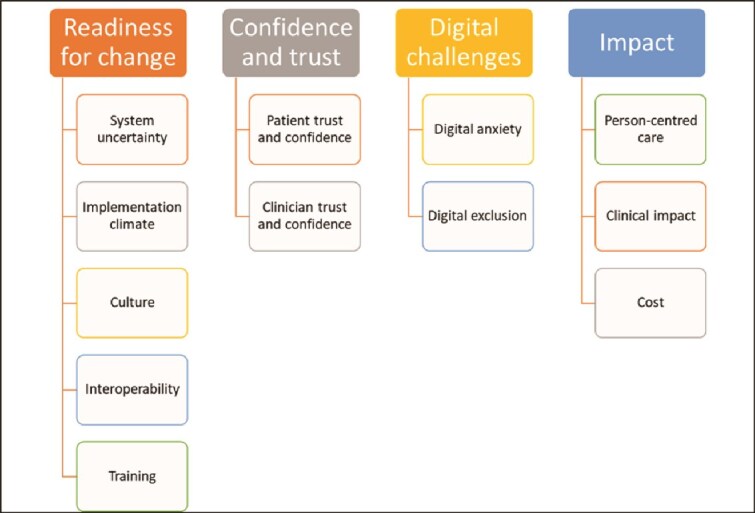
Themes and subthemes

Data were coded to capture its meaning and then clustered into themes and sub-themes where patterns were constructed against the CFIR domains and constructs. This enabled rigorous interpretation and ensured true representation of the meaning of the data. Themes and subthemes are summarized in Fig. 3 and discussed in more detail in the following section. Throughout, verbatim, anonymized quotations are presented to demonstrate how the themes and subthemes emerged from the data.

### Theme 1: Readiness for change

When designing and implementing change in healthcare, being ready to change is vital. The readiness for change theme was developed through the emergence of patterns around healthcare system uncertainty, implementation climate, culture, interoperability and training. It was following the identification of these codes and through further analysis that the established theme was named ‘Readiness for Change’, and this concept was further explored and categorized into five sub-themes of system uncertainty, implementation climate, culture, interoperability and training.

#### Subtheme: System uncertainty

System uncertainty refers to the inherent unpredictability and complexity within healthcare systems when implementing change [8]. It arises from various factors, such as the interdependence of multiple components, the involvement of diverse stakeholders and the dynamic nature of healthcare environments. System uncertainty can present challenges and risks during change implementation. In their interviews, clinicians reported that there was a lack of rigorous evidence and national policies to support virtual ward design and the use of continuous monitoring. There was some uncertainty about continuous monitoring with a view that intermittent monitoring may be superior.

Clinician 10: *‘Yeah, I think with (intermittent monitoring) the data is more accurate’.*

Clinician 39: *‘The virtual ward, national arena, medics, etcetera that are complete converts. But it just feels that we’re lacking in a bit of local and national body guidance’.*

Clinician 40: *‘So, it feels like there is conflicting national… (evidence of) what a good model is’.*

Referral criteria for the virtual wards were evolving and there was some uncertainty around the ‘correct’ patient to admit and concern about a lack of cross-organizational sign-up to using the virtual ward. There were specific challenges over the provision of medical cover and, consequently, a perceived lack of escalation processes for clinicians who were concerned about patient deterioration.

Clinician 34*: ‘Yeah, I think as well because we don’t have our virtual ward medical cover all the time. We are sometimes using the acute GPs and there have been times when they have gone, we’ve escalated, they’ve gone. Why? Why are you escalating it?’.*

In addition, the Cornwall virtual ward model and design was described as unique, as it was community-led with the perception that virtual wards are generally provided by the acute hospital. This led to some uncertainty around the community-led model and its evidence base and a feeling of uncertainty across the whole system, which, in turn, impacted readiness for change.

Clinician 39: *‘Ours (virtual ward) is unique, I think. Being led by community services’.*

Clinician 26: *‘I think, its (Cornwall healthcare) is quite unique. We have many issues and barriers such as three acute trusts, poor internet accessibility. We also have a large portion of complexity within our patients due to the demographic’.*

#### Subtheme: Implementation climate

Implementation climate refers to the organizational environment and culture that supports the successful implementation of change in healthcare settings [9]. It encompasses the attitudes, beliefs and behaviours of individuals within the organization regarding the change initiative [10]. It was in this subtheme that concepts around attitudes, beliefs and behaviours emerged. Clinicians believed there was a strong drive from national policymakers to implement continuous monitoring and that ground-level staff may not have been fully consulted and involved.

Clinician 40: *‘There was significant national pressure and support from NHS England’.*

Clinician 35: *‘...didn’t really understand the continuous monitoring element at all. It was never explained to me, it was more rolled out to us as the fact that we would be having a new system where we would be able to make video calls and the readings would be sent into us’.*

Clinicians identified either a lack of policy guidance or ever-changing national policy intensified by a perceived lack of guidance from national professional bodies. Clinicians felt there was a poor understanding of the realities and risks of running a virtual ward from national leaders and no agreed-upon model at national and local organizational levels. In addition, clinicians identified several rapid major changes within the service happening at the same time. It was felt there was an underestimation of the transformational change needed to implement the new continuous monitoring system. Clinicians spoke about the need for more support around information technology not only from a patient’s perspective but also from a clinician and wider team perspective.

Clinician 08: *‘... we were almost acting like an IT department, and it was a bit different for me personally because I’d seen the equipment I’d gone out and I’d realised what the issues would potentially be…It’s almost like it needed another team that were monitoring the technical side of it’.*

Clinician 26: *‘Yeah, I think I think the technology is too advanced for the structure and current setups of the current of the virtual ward, we just weren’t ready’.*

Communication challenges between the virtual ward and wider community and primary health services were identified with some scepticism about virtual wards, especially from general practitioners, highlighting the need to embed the virtual ward fully into primary and community health services. There were also some challenges around communication among the members of the wider system, which impacted their opinion of the virtual wards.

Clinician 11: *‘I think processes and structures within community services are often seen as barriers, even though they’re implemented with the best intentions’.*

Clinician 34: *‘I think there could be better communication with GPs because we’ve got a patient at the minute where we’ve been titrating meds and doing stuff. And the GPs been doing the same’.*

Virtual ward clinicians felt the service needed to be more visible with active marketing of the lived experience of the patients, leading to a better understanding of the purpose and impact of virtual wards across the whole system.

Clinician 26: *‘… processes and structures that we’ve tried to implement. They just created this negative feedback. So, with us being out and about in person now, the feedback’s positive and now the referrals are coming back in and collaborative working’s happening. We need to share our outcomes’.*

Clinician 39: *‘We have started to share patients’ stories; they people can see the benefits of virtual wards’.*

Despite their concerns, there was a strong recognition and commitment from clinicians that there was a need to do something new and different to change the way people were cared for. They strongly believed that virtual wards were the way forward and recognized their role in promoting the service.

Clinician 40: *‘...we’re really trying to be front and visual and one of our biggest changes of the last 12 months, but we are doing it’.*

Clinician 15: *‘...in the NHS people live in the dark ages that nobody wants any change. And when you try and change something or bring something in, people are wary, aren’t they so? We need to keep going’.*

In summary, there was evidence of barriers and challenges during the initial implementation of the virtual ward and continuous monitoring. However, all clinicians identified the need to continue with the virtual ward model, albeit with a review of processes and procedures and improved sharing of patient outcomes.

#### Subtheme: Culture

Change culture in healthcare refers to the collective beliefs, values and behaviours within an organization that support and encourage the successful implementation of change [9]. It encompasses the attitudes and mindset of healthcare professionals towards change initiatives and their willingness to embrace and adapt to new practices, processes and technologies. By cultivating a positive change culture, healthcare organizations can enhance their ability to adapt to new challenges, improve patient outcomes and drive positive transformation within the healthcare system.

Virtual ward clinicians reported ‘change fatigue’ where there had been multiple changes during and following the COVID-19 pandemic. The rapid implementation of a new system of continuous monitoring within the virtual ward was seen as one change too many. There was a sense that there was an underestimation of the transformational change needed not only within the virtual ward team but also across the wider system and that more support might be needed.

Clinician 38: *‘As a service, we have obviously undergone a huge amount of change over the last year. We continue to undergo huge changes on the backdrop of huge amounts of pressure, over the last 5 years so the team are a bit fatigued’.*

Clinician 31: *‘And I’ve seen all sorts of changes and not all for the better, and I just think it’s just so disjointed. Too much’.*

#### Subtheme: Interoperability

Interoperability refers to the ability of different information systems, devices and applications to seamlessly exchange and use data. When implementing new technologies within healthcare services, interoperability can pose several challenges. Some of the key factors include technical compatibility, data standardization, privacy and security concerns, fragmented systems and legacy infrastructure, governance and policy issues, cost considerations and resistance to change [9]. These challenges were identified by virtual ward clinicians in their interviews, expressing frustration at the lack of compatibility of Information Technology (IT) systems in health care.

Clinician 31: *‘You know, so they could see what we’re about. You know, things like, you know, if you’re worried about someone’s rash sending them a photo, you know, it’d be so much easier if it was just there. You know, we can upload it onto [existing IT system]. But if you’re on the phone to an acute GP, who hasn’t got access, then you’ve got trying to e-mail them a photo and hoping they can get it there. And wouldn’t it be lovely if we just all nationwide have the same system that we could all access? Wouldn’t that be sensible?’.*

Clinician 11: *‘Access to records across trusts and systems can be difficult I guess that’s down to GDPR’.*

Another concern raised was about the continuous monitoring data when inputted into patient National Early Warning Scores (V2) (NEWS2), which are routinely used in the virtual ward to assess the degree of illness and risk of deterioration. The NEWS scores were used to escalate patients who were deteriorating; however, they caused unnecessary concern for the clinicians when they were based on data from the continuous monitoring system. It became clear that the systems were not compatible, and the only way to continue continuous monitoring would have been to stop using NEWS scores. This was not acceptable for the organization regarding patient safety.

Clinician 8: *‘NEWS2 does not work with (continuous monitoring provider) monitoring. Because we are not and will never have a cohort of average adults that sit within normal parameters, what we have are cohorts of patients that are sick’.*

Clinician 10: *‘But I think we’ve run into a few issues with it (continuous monitoring) with the accuracy perhaps of some of the data and how that fits in with the NEWS2 type system that we’re using to monitor patients clinically’.*

In summary, the lack of shared or compatible computer systems caused frustrations and some clinical safety concerns for clinicians in sharing information across organizations and in using data from the continuous monitoring system to populate mandatory clinical deterioration assessments.

#### Subtheme: Training

Implementing training during a rapid innovation service change in healthcare can bring both challenges and benefits [11]. There were some apparent challenges relating to training for clinicians and patients using continuous monitoring within the virtual ward. More training was needed for patients who were discharged from the acute hospital onto the virtual ward. Patients described how they were given little or no information from hospital staff about their admission to the virtual ward or how to use the monitoring equipment they were given. This necessitated extra support from the virtual ward team and even some home visits if the patient needed further support.

Patient 3: *‘Because I wasn’t told how to do it. Basically, only the chap turned up that day. Well, I was still in bed’.*

Patient 24: *‘Well, yeah, instead of being given two boxes of tablets and a bag, a sealed bag, appearing and nobody else coming along to say, “By the way, this is…” I had no idea whatsoever what was going on’.*

Clinicians identified the need for more training on how to interpret data provided by the continuous monitoring system. They needed well-developed clinical reasoning skills to interpret the data accurately and not all felt safe in making judgements based on the information provided. Most clinicians felt that more training was needed on using the continuous monitoring system but, most importantly, on how to interpret that data effectively. In addition, they wanted this to be ongoing training, not just at the implementation phase.

Clinician 38: *‘They never (Continuous Monitoring Provider) never really gave us any information about interpreting the data. There was never any real training around that, and I think that sort of data isn’t used in the NHS’.*

For the future, clinicians recommended that digital health training be included in pre-registration training for nurses, doctors and allied health professionals so that there is a workforce that leaves training with the skills to specialize in this area and work in the virtual environment. In addition, it was proposed that there could be continuing professional development modules available for those whose original pre-registration course did not include digital health or for those needing an update.

Clinician 40: *‘Particularly when maybe students are out on visits or on the wards and you know if they feel strong enough to suggest to their supervisors or mentors, then I think that’s positive. There’s a lot of master’s education about digital health services and digital health, nursing and digital health leadership. I would welcome specific modules at Uni. On digital health nursing’.*

Overall, whilst clinicians received initial training, it was felt that this could have been more in-depth and included support to interpret data. There was also evidence of a wider need for education for clinicians across disciplines by including digital health in pre-registration curricula.

### Theme 2: Confidence and trust

The concepts of confidence and trust were identified by patients, clinicians and service managers in their interviews. Confidence and trust in digital healthcare settings refer to the belief and reliance that individuals have in the security, accuracy and effectiveness of using digital technologies for healthcare purposes [12]. It involves having trust in the confidentiality of personal health information, the reliability of digital health tools and services and the competency of healthcare professionals who utilize these technologies [13]. This theme was sub-divided to explore these concepts relating to patients and clinicians.

#### Subtheme: Patient confidence and trust

Feeling reassured and safe with confidence in the virtual ward clinicians was a strong theme in the patient interviews. Most patients interviewed identified that this was linked to the fact that someone was monitoring them, and they could get help if things went wrong. This was strongly appreciated.

Patient 10: *‘It was kind of reassuring to speak to somebody on a daily basis...and to know that I wasn’t just sitting there going downhill’.*

Patient 4: *‘The nurse gave me a number and I could ring up the virtual ward and they would help sort it out’.*

Patient 3: *‘I feel very secure at home. Confident that someone was there’.*

In addition, patients valued the kindness, compassion and professionalism of the virtual ward clinicians they encountered. Patients and carers suggested the level of care they received, matched and even exceeded the care they received in hospital and that they could rest and recover more effectively at home. Consequently, they felt more confident to remain safely at home and manage their condition with the support of the virtual ward team.

Patient 16: *‘But that gave me the boost that I needed that there are still people out there that really do care and love their job and it’s… I can’t explain what I’m trying to say. It just makes such a difference to someone’s life… I’ve got nothing but praise for them, all of them. They were excellent’.*

Patient 8: *‘Well, I… I was glad to be home in many respects, because the… the noise at night (in the hospital) m was horrendous’.*

Patient 12: *‘...I’ll tell you what, I’d use that (Virtual Ward) again sooner than go into hospital’.*

Some patients experienced no difficulties with using any of the remote monitoring equipment and found they valued certain features such as instant messaging. Having instant access to a clinician was highly valued and added to confidence and trust.

Patient 8: *‘you can actually click a button and then instantly you can message them as well on the side. But you can instantly visually see them. So that’s also a safety aspect’.*

Patient 16: *‘It was a week. I wish it had been longer really because I felt safe… and for that week I felt comfortable in my own shoes. I felt the support was there. Any questions were answered and just someone, that phone actually going and someone enquiring, not that I’m… it’s attention’.*

However, some patients did identify challenges to using some of the equipment provided involving the wearable as well as the tablet devices. Some patients with poor dexterity struggled to apply the wearable device, and others had difficulty remembering how to apply the device and how to use the tablet.

Patient 3: *‘It was all right, but I was a little bit confused about using it. I just couldn’t remember’.*

Patient 4*: ‘A little bit putting the pad on to take my blood pressure because I have trouble with hands. My hands are quite numb. I didn’t want to keep asking him can you do this; can you do this? But I did manage. If they don’t get a proper reading, they let me know, but I did manage it but it was a little bit tricky because of my fingers’.*

Overall, the patients identified benefits and challenges, which impacted on their confidence and trust in the technology and systems. The easily accessible contact with a clinician provided by the virtual ward gave reassurance and improved confidence.

#### Subtheme: Clinician trust and confidence

Previous experience, knowledge and skills underpinning the clinician’s belief in their capacity to effectively support the patients appeared to have a direct correlation to their confidence and trust in themselves and the service. Clinicians with a lack of experience in this type of consultation appeared to struggle to assess and interpret the continuous monitoring data. This affected trust in the reliability of the continuous monitoring data.

Clinician 10: *‘But I think we’ve run into a few issues with it with the accuracy perhaps of some of the data’.*

Clinician 34: *‘You know, unreliable data coming through continuously when it was 15 minutes behind as well. So, you’re on the phone with someone. Then they (oxygen saturation levels) are 87, but you’re looking at them and they’re pink. They’re not short of breath. There are no worrying things’.*

Clinician 39: *‘Never really gave us any information about interpreting the data. There was never any real training around that...’.*

The comments above were made at a time early in the implementation of continuous monitoring relating to the incompatibility of the continuous monitoring data with existing clinical procedures including the use of NEWS scores to assess patient deterioration. These experiences had impacted the clinician’s trust and confidence in the technology and their ability to use it effectively in patient management. This then led to a wider problem of concerns about clinical safety and the accountability of the nurses who were receiving and interpreting data from the continuous monitoring system.

Clinician 34: *‘You know the nurses’ PINs (Nursing and Midwifery Council Professional Identification Numbers) are at risk…if you took the NEWS from (Continuous Monitoring Equipment Provider) and then you took the NEWS from the spot monitoring, they could be a nine on (Continuous Monitoring Equipment Provider), but then they could be six on spot monitoring’.*

Clinician 30: *‘Which means that we get patients that are NEWS’sing at eight at nine. And it’s generating work. It’s generating anxiety not just for the staff, but for the patient as well, because then they’re getting constant contact because the monitoring is there from 8:00 o’clock in the morning till 6:00 o’clock in the evening. So, every time they spike in, the alarm goes off. We have to action’.*

Despite their concerns, some clinicians stated that if they had more time to get used to the continuous monitoring system, they could envisage how useful the data and other aspects of the system might be.

Clinician 39: *‘So it gave the information a very different context that we were not used to dealing with, and I think fundamentally that’s why it didn’t work is you need to train the staff how to interpret that data in a different way and how to understand what that data is because people didn’t understand the data it made everybody think that it wasn’t accurate. Had we had more time, I think there would have been a different result’.*

Clinician 26: *‘So, the (Continuous Monitoring Equipment Provider) video calling was phenomenal. It was a game changer. I assessed the patient clinically, you know CRT sign, you know looked to the accessory muscles, respiratory rate, the quality of the picture was good’.*

In summary, patients expressed high levels of confidence and trust due to the reassurance provided by the virtual ward monitoring and easy access to professional, compassionate clinicians. However, clinicians lost confidence in the continuous monitoring system mainly because of its incompatibility with their existing clinical procedures and lack of understanding of how to interpret the data safely. Nonetheless, they could see the advantages of continuous monitoring and with more time and training felt that it could be a useful tool within the virtual ward setting.

### Theme 3: Digital challenges

Whilst the digital health revolution offers numerous benefits, it also presents some challenges including interoperability difficulties, concerns over data privacy and security, digital exclusion and anxiety, ensuring the presence of appropriate regulatory and legal frameworks and data accuracy and reliability [14, 15]. These challenges were reported in the patient and clinician interviews and categorized into the sub-themes of digital anxiety and digital exclusion.

#### Subtheme: Digital anxiety

Digital anxiety refers to the stress, unease or fear that individuals may experience when using or interacting with digital technologies. It is a psychological response that can arise from various factors related to digital tools and platforms [16]. Digital anxiety can have a negative impact on individuals health and wellbeing, and this resonated with both clinicians and patients in their interviews. Clinicians spoke about the difficulties some patients experienced and particularly how the equipment raised anxiety, predominantly if it was too complicated to use. This was also seen in the patient responses, especially how design issues caused anxiety when using the technology. For example, patients reported that you could not tell whether the equipment was switched on and how they worried they may not have been transmitting data to the virtual ward team.

Patient 17: *‘I just never knew if it was on, I mean would it send information’.*

Clinician 38: *‘Some patients, with the continuous monitoring were freaking out as soon as you mentioned a tablet and that was before it was even delivered. They were just... “I don’t even use a mobile phone”’.*

In addition, some patients reported that having a large amount of equipment was daunting, particularly those who were sent home from hospital with the equipment and given little explanation on how to use it. Having said that, patients who had experienced both intermittent and continuous monitoring felt that their anxiety lessened with continuous monitoring, and this was due to not having to apply and remove the wearable device frequently.

Clinician 15: *‘Maybe more information in the hospital before patients are discharged, somebody to go round wherever they’re discharged from and really go through the equipment with them and really explain because I think we find that sometimes patients get discharged onto our ward and you’ll ring them up and they haven’t. They don’t even know they’ve consented to it for starters, that the monitoring and then they’re like, oh, OK, has anyone been through the kit with you? Oh, I don’t remember. I can’t. I can’t think or they’re just very flustered when they get home. And I think we need to be giving them the easiest option’.*

Clinician 34: *‘For some of those older people compared to our virtual ward spot monitoring where you’ve got two hands to do it or, you know, have a bit of a fight to adjust it, that was quite good. I think that was helpful for some’.*

As discussed in the theme of trust and confidence, clinicians identified their anxiety around the frequency, interpretation and escalation of the continuous monitoring data. In addition to concerns about interpreting data and potential inaccuracies, they expressed concern that frequent alarms sent by the system when patient parameters were breached caused anxiety.

Clinician 38: *‘You were literally minute by minute……… which did throw up a lot of anxiety. This end because you’ve got a patient who’s a respiratory patient. They would desaturate and you’d be like. OK. you’d be like…. What are you doing? You’d find yourself making stories in your heads because to, to be honest, when we rang people, they were often on the toilet’.*

#### Subtheme: Digital exclusion

Digital exclusion refers to unequal access and participation in digital technologies and the internet. It describes the gap between those who have access to digital resources and those who do not, leading to disparities in knowledge, skills and opportunities [16]. Digital exclusion is multifactorial and can occur at various levels, including individuals, communities and even entire countries [17]. The factors that can result in digital exclusion include access to technology, limited digital literacy, affordability and language, age and cultural barriers [16]. Both clinician and patient interviewees referred to these factors as potential barriers to using technology in the virtual ward. Some patients were excluded from using the monitoring equipment due to internet connection difficulties. This is a common theme within telehealth and virtual wards generally [18]. There were specific connectivity issues with the wearable devices used in continuous monitoring, and this did raise anxiety for some clinicians. In addition, body habitus and tattoos led to difficulties in applying and gaining data from the wearable devices.

Clinician 14: *‘But for whatever reason, the way the data was coming to us, seemed to stop, not sure why’.*

Clinician 38: *‘...you know, we had a problem with a guy over tattoos on both arms and it didn’t work on him. We had a lady that was too small, so the cuff didn’t work on her’.*

Patient 10: *‘Bad reception on a couple of occasions...’.*

In summary, aspects of digital anxiety were expressed by clinicians and patients. In addition, there were examples of digital exclusion, due to body habitus, understanding of the technology and connection difficulties.

### Theme 4: Impact

The impact of a health service can be measured in various ways, such as health outcomes, access to care, cost-effectiveness, user satisfaction and system sustainability. These concepts emerged from both patient and clinician interview data and categorized into three sub-themes of person-centred care, cost and clinical impact.

#### Subtheme: Person-centred care

Person-centred care is an approach to healthcare that prioritizes the individual’s needs, preferences and values. It recognizes that each person is unique and should be actively involved in their own healthcare decisions and treatment planning [19]. There are key principles of person-centred care, including respect for individuality, collaboration and shared decision-making, empathy and compassion, continuity and coordination of care, taking a holistic approach and individualized care planning. The importance of these concepts was cited by clinicians and patients in their interviews. This is positive, as person-centred care has been shown to improve patient satisfaction, engagement, adherence to treatment and health outcomes [20]. Clinicians and patients alluded to person-centred care when they identified how they were providing or receiving ethical healthcare and perceived that they were enabling or receiving informed choice. This included offering care in the virtual ward as a real alternative to hospital admission. Patients reported that this shared decision fitted with their lifestyle, and they talked about being scared to go into hospital since the COVID-19 pandemic. Admission to the virtual ward provided a real alternative for them.

Clinician 40: *‘And I think virtual wards actually gifts us the ability to provide truly personalised care for the first time in a long time’.*

Clinician 40: *‘Say its 1600 o’clock on a Friday evening, other than to convey somebody to hospital, now there’s the option and actually being able to have that really honest discussion with the patient that if we convey you tonight, this evening it’s very likely you may be sitting on the back of an ambulance for a few hours, then you will be in ED for a few hours. Then you’ll be on another ward. Or we can support you to stay at home using virtual ward to monitor you. You will have a medic with you. You will have community services going in to support you if you need to have a specific diagnosed diagnostic that cannot be done in the community, we can arrange that as an outpatient. What? What would you like to do?’.*

Patient 15: *‘Yeah. But, no, if it wasn’t for the virtual ward I guarantee I would have been back in hospital. So, it saved me from that’.*

Conversely, some patients did identify areas where person-centred care was initially negatively impacted; however, once clinicians and patients became familiar with the virtual ward processes, they were able to personalize the individual’s care.

Patient 15: *‘I used to say it felt as if I was being confined to the house, couldn’t go out anywhere or do anything, because I did it on the first day and everybody panicked because they said I’ve gone off radar and I said, “‘I’m sorry, I didn’t realise.” But if I told them prior then it was, they wouldn’t panic so much, but I only did it a couple of times’.*

In general, the person-centred approach taken by the virtual ward clinicians was valued and led to high levels of patient satisfaction.

#### Subtheme: Cost

When evaluating a service, it is important to analyse its cost-effectiveness both in relation to resource allocation, affordability, sustainability, efficacy and waste reduction well as cost-effectiveness [8]. In the interviews, there was evidence of consideration of the wider cost implications of virtual wards with patients and clinicians discussing both monetary and social costs. The majority of patients had not considered any additional costs to them or their families associated with virtual ward admission. They did report, however, how it saved some costs associated with hospital admission.

Patient 4: *‘Costs, No, I… I didn’t… didn’t come into the equation, actually, at all’.*

Patient 24: *‘Not really, no. At the end of the day, it would cost a lot more for my family to come down and visit me in hospital…You know, for what those costs, it must be piddly, you know’.*

Of the patients who had considered costs, they felt costs of operating the remote monitoring equipment were minimal or necessary for the care they were receiving from the virtual ward.

Patient 11: *‘No, I… I switched it off at night…And I just… I just viewed it as a necessary evil, the cost of running…The cost of running must be minimal anyway’.*

There were a variety of financial impacts identified by clinicians, both positive and negative. Many clinicians felt virtual wards were cost-saving for the organization and wider health system. They spoke about the potential environmental benefits of a reduction in car travel to visit the patients at home. It was felt there was a significant saving of acute hospital beds and cost savings across the whole system, although it was acknowledged that this was challenging to quantify. Conversely, other clinicians identified potential financial costs to the community health organization, an example was providing transport for patients to attend outpatient X-ray if this is needed for patients in the virtual ward. In addition, there was identification of costs of home visits where virtual ward team members visited patients to troubleshoot any technical issues.

Clinician 8: *‘I would say it’s not cost effective because we’re sending staff out, we’re sending new equipment out, we’re having to double up on what we’re using. They’re getting more visits; more contact because we’re having to double check everything’.*

Clinician 11: *‘...the idea is to prevent this hospital admission, this hospital bed I think in you know a couple of phone calls most of the time now everyone has unlimited minutes, and you know there’s plans as phone call plans so using phone call as an example it would definitely always be less expensive than having to have a patient physically on a hospital bed’.*

Clinician 40: *‘From a qualitative and social perspective, the benefits I think are high as well as from a financial perspective’.*

In summary, patients perceived there was no cost to them or that virtual wards were cost saving. Clinicians believed that a virtual ward could be cost saving to the patient and the health service but that more evidence was required to effectively demonstrate cost benefit.

#### Subtheme: Clinical impact

Clinical impact refers to the effect or influence that a healthcare intervention, treatment or service has on patient outcomes, health status and overall well-being [8]. This final subtheme concerns the views of both patients and clinicians on the clinical impact of the virtual ward. Patients compared how simple it was to contact the virtual ward and compared it with how difficult it could be to make an appointment to see a general practitioner, allowing improved access to primary healthcare.

Patient 8: *‘I do. I’ll tell you what, if they use… well, put it this way, I could never get to see my doctor already or anything and while I’ve got that, if I was a bit confused about anything, I could ring up the virtual ward and they would help sort it out’.*

Clinicians identified clinical advantages and concerns around the introduction of continuous monitoring. The clinical advantages of continuous monitoring were identified, even though it was discontinued early. This was particularly apparent for the senior nurses who stated that continuous monitoring provided more detailed patient data than intermittent monitoring. Data were received in a timely manner, thus aiding their clinical decision-making. In addition, the presentation of the data on the system enabled them to scan across biometrics for all patients in the virtual wards. This was visually easier for the senior nurses having to prioritize their response to patient need.

Clinician 11: *‘I think for both patients. And I think for even us clinicians, we can get the information we need real time. We can make decisions except for obviously you’ll just a few cases where, you know, cause it’s not obviously it’s not 100%, its technology. So, it’s gonna have its drawbacks. But on the whole, we can make rapid decisions’.*

Clinician 15: *‘...personally I know there’s issues with (Continuous Monitoring), but I personally preferred it having used that for a couple of weeks. I thought, oh, this is actually a good system being a band 6 overlooking the ward, you can see things that quickly rather than waiting for people to ring in and then give you the information then escalate to you, you’ve got a better overview...so, it’s like visually seeing the whole of your ward on one screen, which I find quite a better system than spot monitoring’.*

Clinicians were able to identify potential serious problems or underlying conditions, using 24-hour data collection provided by the continuous monitoring equipment. Some clinicians identified that access to a 24-hour monitoring period was useful, even though the virtual ward does not operate overnight. Having a view of the patient’s biometrics over a 24-hour period aided clinical decision-making.

Clinician 38: *‘I think that’s I think the data was useful seeing if they were desaturating, we only used it for 12 hours of the day. There was one lady that kept it on all night that we were able to see that she was desaturating quite a bit at night, even though we’re not supposed to be looking at it because she had it on. You could actually see that while she was sleeping’.*

Clinicians also saw the benefit of contact provided by instant messaging and video calls. Combined with the continuous monitoring data, they could assess how that person looked face to face.

Clinician 15: *‘Whereas on the (Continuous Monitoring System) ...you can actually click a button and then instantly you can message them as well on the side. But you can instantly visually see them. So that’s also a safety aspect’.*

Overall, the impact of continuous monitoring was impaired due to the challenges with the utilization and interpretation of the data and its use was discontinued after a short time in with virtual wards in Cornwall. Despite this, it does appear that there were positive clinical and patient impacts from the use of continuous monitoring within the virtual ward setting. Many of the negative impacts could be overcome by reviewing the type of technology used and this is discussed further in the next section.

## DISCUSSION

The development of virtual wards in Cornwall, UK, progressed rapidly during the COVID-19 pandemic, necessitating swift adaptations as the pandemic subsided. This led to some implementation issues compounded by change fatigue when the new continuous monitoring system was introduced. As the CFIR Framework had been used to structure the evaluation, it was possible to adapt and enable a broader focus that examined the functioning of the virtual ward within the organization in the context of the broader healthcare system. The use of the CFIR domain relating to implementation characteristics enabled identification of and exploration of the specific implementation characteristics of the Cornwall virtual wards (Fig. 2). The ‘innovation source’ construct was explored with clinicians, gaining their perspectives on the need for the new continuous monitoring system and their concerns about a lack of underpinning good-quality evidence base. The construct regarding ‘relative advantage’ of the new system was explored, and concerns about the system’s ‘adaptability’ to the virtual wards were discussed. Considering the construct of ‘design, quality and packaging’ highlighted the importance of adequate training and resources to support implementation and the construct of ‘cost’ shaped data regarding hidden costs and the potential for cost-savings with effective implementation.

The rapid adoption of the new continuous monitoring system led to staff feeling unprepared for the change and important stakeholders not fully engaged. These findings concur with the WHO report [2] that identified health system challenges in the implementation of digital health solutions including insufficient workforce competence/supervision, inadequate identification and management of risks and lack of wider system engagement. This evaluation highlighted the concerns of virtual ward clinicians regarding a lack of evidence for the most effective virtual ward models, types of monitoring and guidelines to support practice, and further research in these areas is required. Other issues could be addressed with a more structured approach to implementation with a stakeholder engagement exercise across the whole system and an assessment of readiness to change using a validated tool. This should include the identification of change agents, individuals or groups that play a central role in facilitating and driving the change process. These change agents are central to raising awareness among staff of the national and international evidence relating to virtual wards and working with patients and other champions in the co-production of a new virtual ward model. In addition, virtual ward staff should be supported to deal with change by provision of wellbeing support, clinical supervision, appraisal and other measures such as flexible working.

Despite the concerns and challenges highlighted by the virtual ward clinicians, the overwhelming response from patients demonstrated how much they valued the virtual ward as a viable alternative to hospital admission. The compassion and professionalism shown by the virtual ward clinicians made patients feel safe and cared for in their own homes. Whilst undoubtedly, the presence and use of monitoring equipment contributed to these feelings of safety, the ability to be able to contact the virtual ward clinicians for support and to know that someone was there monitoring their condition were key factors that made this a viable alternative to hospital admission for the patients. Whatever technology is used in a virtual ward model, it is the interaction with the staff that is highly valued and leads to enhanced patient confidence to manage their conditions out of hospital. It is, therefore, vital that the staff are well trained, supported and able to develop their skills further.

Looking to the future, there is a need to consider the sustainability of the project beyond the implementation phase using ongoing audit and continuous quality improvement, scaling up and resource allocation. De la Boutetiere *et al*. [21] reported that investing in digital technology frequently yields minimal returns and does not guarantee improved performance or effective embedding within existing services. Marikyan and Papagiannidis proposed a ‘Unified Theory of Acceptance and Use of Technology’ (UTAUT) [21]. They proposed that the utilization of technology is governed by individuals’ behavioural intentions determined by four key concepts of ‘performance expectancy, effort expectancy, social influence and facilitating conditions’ [22]. Our findings concur with this theoretical approach, demonstrating that clinicians’ perceptions of whether using the technology will improve their job role, its’ perceived ease of use, its relative importance within the wider healthcare system and their beliefs about the organization’s technical infrastructure ability to support the use of the new system are crucial factors in implementation success. In addition to the UTAUT proposals, our findings highlight the need for effective planning of implementation, adequate training both initially and on an ongoing basis and assessment of compatibility with existing technologies.

The virtual ward model needs to be fully embedded in mainstream healthcare services with a focus on strategies that will enhance the change process by addressing the specific learning points identified. These include working with community healthcare colleagues, developing a robust medical support model, addressing IT usability and connection challenges, raising public awareness of virtual wards and providing support and education for referrers to increase understanding and confidence in using the virtual ward. Further research is required to validate any perceived financial or environmental benefits.

### Limitations

There are several limiting factors in this service evaluation, mostly due to the significant change made when it was decided that the use of the continuous monitoring equipment would be discontinued after a very short period. This meant that patients and clinicians interviewed had limited experience with continuous monitoring; therefore, it was not fully possible to meet the original objective of comparing continuous with intermittent monitoring. Despite this challenge, the use of the CFIR Framework enabled us to gain a much broader understanding of the virtual ward and its development, so the changing focus led to a more robust and overarching service evaluation.

## CONCLUSION

Virtual wards are becoming established globally, and their inception and development have happened at pace. As evidenced by this service evaluation, this rapid evolution can impact staff and service delivery, but it is important to acknowledge that these challenges are intrinsic in post-pandemic times and the associated innovative digital solutions that have emerged. Further research is now required into how models of virtual wards that are effective and sustainable can be achieved and maintained.

## Supplementary Material

Supplementary_Materials_oqaf008

## Data Availability

The data underlying this article are available in the article and in its online supplementary material.
