# Supplementary material for: A service evaluation of virtual wards in Cornwall, UK
Source: Oxf Open Digit Health. 2025 Apr 14;3:oqaf008. doi: 10.1093/oodh/oqaf008 (PMC12053098; doi:10.1093/oodh/oqaf008)
Supplement: Supplementary_Materials_oqaf008 [file supplementary_materials_oqaf008.docx]

# Supplementary Materials: Interview Guides

A Service Evaluation of Virtual Wards in Cornwall, UK.

## Author Affiliations

*Helen Lyndon ^1,2^

Tracey Viney ^3^

Vicki Slade ^2^

1. University of Plymouth, South West Clinical School, Plymouth, Devon, UK
2. Cornwall Partnership NHS Foundation Trust, Digital Health Service, Bodmin, Cornwall, UK
3. Marjon University, School of Health and Wellbeing, Plymouth, Devon, UK

## Corresponding author

Dr Helen Lyndon

Cornwall Foundation NHS Trust, Green Court, Threemilestone, Truro, TR4 9LF, Cornwall, UK.

Email: [helen.lyndon@plymouth.ac.uk](mailto:helen.lyndon@plymouth.ac.uk)

# Interview Guide Patients

# 1.0 INNOVATION DOMAIN

**Introductory question:**

Tell me about your experience of being on the virtual ward.

**Prompts:**

- What did you like about being on a virtual ward?
- What did you dislike about being on a virtual ward?
- What were the advantages/disadvantages for you and your family/carers?
- Did anything cause you concern or worry you?
- How easy did you find using the system?
- How easy was the equipment to use?
- Did you experience any difficulties with using the equipment e.g., internet access, applying the devices, contacting the virtual ward team?
- Did you feel you could access help when you needed it?
- If you became unwell again, would you want to be admitted to the virtual ward?

**For patients who have experienced both continuous and intermittent monitoring:**

Which type of monitoring did you find the easiest to use?

Why was that?

# 2.0 INNER SETTING DOMAIN

Were there any financial implications for you in being on the virtual ward?

- Any additional costs such as light, heating, equipment?

Is there anything else you would like to tell us about your experience of being on the virtual ward?

Interview Guide Clinicians

1.0 INNOVATION DOMAIN

1.1 Intervention Source

1. Why is continuous monitoring being implemented?
2. Who decided to implement continuous monitoring?
3. How was the decision made to implement continuous monitoring?

1.2 Evidence Strength & Quality

1. What kind of supporting evidence or proof is needed about the effectiveness of continuous monitoring to get staff on board?

1.3 Relative Advantage

1. How does using continuous monitoring compare to intermittent monitoring in the virtual ward?
2. Tell me about your experience of caring for patients using continuous monitoring?

- What are the advantages/disadvantages?
- How does it support clinical decision-making?

1. Tell me about your experience of caring for patients using intermittent monitoring?
2. What are the advantages/disadvantages?
3. How does it support clinical decision-making?
4. Have you had experience of using both intermittent and continuous monitoring?

- If yes, how do they compare?
- Advantages/disadvantages/ease of use/quantity and quality of data provided by each system?

1.4 Adaptability

1. What kinds of changes or alterations do you think you will need to make to so intermittent monitoring will work effectively in the virtual ward for the future?
2. Do you think you will be able to make these changes?

- Why or why not?

1. Are there components that should not be altered?
2. Which ones should not be altered?

1.5 Complexity

1. How complicated is continuous monitoring compared to intermittent monitoring?

- Consider the following aspects: duration, scope, intricacy, and number of steps involved and whether the intervention reflects a clear departure from previous practices.

1.6 Design Quality & Packaging

1. What supports, such as online resources, marketing materials, or a toolkit, are available to help you implement and use continuous monitoring?
2. How do you access these materials?

1.7 Cost

1. What costs will be incurred to implement continuous monitoring?

- Are there any hidden costs, such as additional clinical time?

1. Does continuous monitoring have the potential to make any cost savings?

2.0 OUTER SETTING DOMAIN

2.1 Patient Needs & Resources

1. How well do you think continuous monitoring meets the needs of patients in the virtual ward?
2. In what ways will the continuous monitoring meet their needs?

- For example: improved access to services/reduce digital exclusion? Help with self-management? Reduced travel time and expense?

1. How does this compare to intermittent monitoring?
2. How do you think the patients will respond to using continuous monitoring?
3. What barriers will patients face to using continuous monitoring?
4. Have you heard stories about the experiences of patients using continuous monitoring?
5. Can you describe a specific story?

2.2 External Policies & Incentives

1. What kind of performance measures, policies, regulations, or guidelines influenced the decision to implement continuous monitoring?
2. How will you be using continuous monitoring help CFT to meet these measures, policies, regulations, or guidelines?
3. What kind of financial or other incentives influenced the decision to implement continuous monitoring?

3.0 INNER SETTING DOMAIN

3.1 Structural Characteristics

1. What kinds of infrastructure changes will be needed to accommodate continuous monitoring for the future?

- Changes in scope of practice? Changes in formal policies? Changes in information systems or electronic records systems? Other?

1. What kind of approvals will be needed? Who needed to be involved?
2. Can you describe the process that will be needed to make these changes?

3.2 Tension for Change

1. Is there a strong need for using intermittent monitoring?

- Why or why not?

1. Do others see a need for it?
2. How essential is this change to meet the needs of patients or other organisational goals and objectives?
3. To what extent does intermittent monitoring fail to meet existing needs? Will the continuous monitoring meet these needs?
4. How will the continuous monitoring fill current gaps?

3.3 Compatibility

1. How well does continuous monitoring fit with existing work processes and practices in the virtual ward?
2. What are likely issues or complications that may arise?
3. Can you describe how continuous monitoring will be integrated into current processes?
4. How does it interact or conflict with current programs or processes?
5. Could continuous monitoring replace or compliment intermittent monitoring in the virtual ward?

4.0 CHARACTERISTICS OF INDIVIDUALS DOMAIN

4.1 Knowledge & Beliefs about the Intervention

1. Do you think continuous monitoring is effective in the virtual ward?

- Why or why not?

1. How do you feel about the continuous monitoring being used in the virtual ward?
2. Do you have any feelings of anticipation? Stress? Enthusiasm? Why?
